# A curated transcriptome dataset collection to investigate the immunobiology of HIV infection

**DOI:** 10.12688/f1000research.8204.1

**Published:** 2016-03-11

**Authors:** Jana Blazkova, Sabri Boughorbel, Scott Presnell, Charlie Quinn, Damien Chaussabel

**Affiliations:** 1 Sidra Medical and Research Center, Doha, Qatar; 2 Benaroya Research Institute, Research Technology, Seattle, WA, USA

**Keywords:** Transcriptomics, Bioinformatics, Software, HIV, Immune Response, Big Data

## Abstract

Compendia of large-scale datasets available in public repositories provide an opportunity to identify and fill current gaps in biomedical knowledge. But first, these data need to be readily accessible to research investigators for interpretation. Here, we make available a collection of transcriptome datasets relevant to HIV infection. A total of 2717 unique transcriptional profiles distributed among 34 datasets were identified, retrieved from the NCBI Gene Expression Omnibus (GEO), and loaded in a custom web application, the Gene Expression Browser (GXB), designed for interactive query and visualization of integrated large-scale data. Multiple sample groupings and rank lists were created to facilitate dataset query and interpretation via this interface. Web links to customized graphical views can be generated by users and subsequently inserted in manuscripts reporting novel findings, such as discovery notes. The tool also enables browsing of a single gene across projects, which can provide new perspectives on the role of a given molecule across biological systems. This curated dataset collection is available at: http://hiv.gxbsidra.org/dm3/geneBrowser/list.

## Introduction

Uncovering the gene transcription signature associated with different outcomes of HIV infection is paramount to a deeper understanding of HIV pathogenesis and to identifying potential therapeutic targets for improving immunological response and for eradicating HIV infection ^1^. HIV has a complex life cycle during which it engages multiple host cellular components, including the immune cells in which it replicates, undermining immune functions. It also highjacks host transcription factors and enzymes to assure viral production and subsequent infections ^2^. HIV dysregulates host genes resulting in aberrant immune response, disease progression, and opportunistic infections ^3, 4^. The ability to pool and analyze samples across various groups of HIV infected individuals with different disease outcomes and across various cell types or tissues, offers a unique opportunity to define common denominators of the immune control of HIV infection, the regulation of HIV replication, and/or the virus-host interaction. With this in mind, we make available, via an interactive web application, a curated collection of transcriptome datasets relevant to HIV infection.

With over 65,000 studies deposited in the NCBI Gene Expression Omnibus (GEO), a public repository of transcriptome profiles, the identification of datasets relevant to a particular research area is not straightforward. Furthermore, GEO is primarily designed as a repository for storing data, rather than for browsing and interacting with the data. Thus, we used a custom web application, the gene expression browser (GXB), to host a collection of datasets that we identified as particularly relevant to the study of the immunobiology of HIV infection. This tool has been described in detail and the source code released as part of a recent publication ^5^. It allows seamless browsing and interactive visualization of large volumes of heterogeneous data. Users can easily customize data plots by adding multiple layers of information, modifying the sample order and generating links that capture these settings and can be inserted in email communications or in publications. Accessing the tool via these links also provides access to rich contextual information essential for data interpretation. This includes for instance access to gene information and relevant literature, study design, and detailed sample information.

## Material and methods

### Identification of relevant datasets

Potentially relevant datasets deposited in GEO were identified using an advanced query based on the Bioconductor package GEOmetadb, version 1.30.0, and on the SQLite database that captures detailed information on GEO data structure ( https://www.bioconductor.org/packages/release/bioc/html/GEOmetadb.html) ^6^. The search query was designed to retrieve entries where the title or summary contained the word HIV, and were generated from human samples using Illumina or Affymetrix commercial platforms.

**Figure 1.**
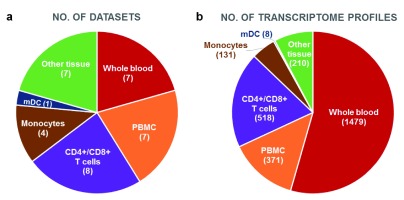
Sample source composition of the dataset collection. Pie charts representing the numbers of datasets ( **a**) or transcriptome profiles ( **b**) for different cell types and tissues.

**Figure 2.**
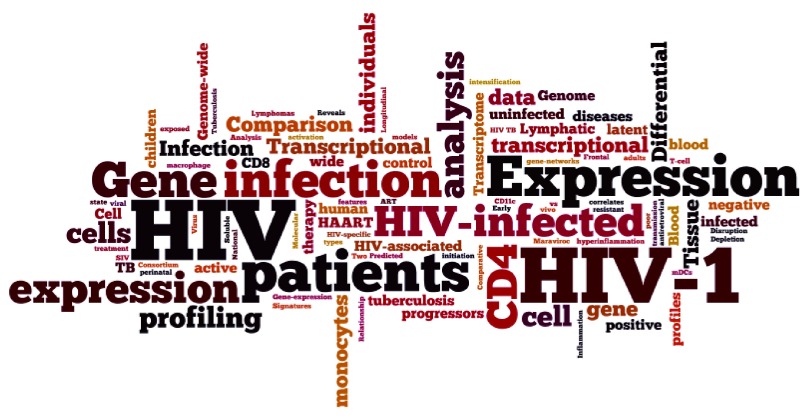
Thematic composition of the dataset collection. Word frequencies extracted from titles of the studies loaded into the GXB tool are depicted as a word cloud. The size of the word is proportional to its frequency.

The relevance of each entry returned by this query was assessed individually. This process involved reading through the descriptions and examining the list of available samples and their annotations. Sometimes it was also necessary to review the original published report in which the design of the study and generation of the dataset are described in more details. We identified 87 datasets meeting the search criteria and containing HIV infected samples (some studies related to HIV problematics contained uninfected samples only). Out of the 87 datasets, 41 were generated from tissues or cells isolated from HIV infected individuals, 46 contained cell lines or primary cells infected *in vitro*. Since molecular, cellular and physiological processes involved in the context of *in vivo* and *in vitro* infections are dramatically different, we decided to create two separate collections. Here we describe the “ *in vivo* collection” composed of 34 curated datasets (after filtering out datasets that did not meet quality control criteria, as described in “Dataset Validation” section, or datasets generated using an unsupported array platform). Of the 34 datasets, 7 are from whole blood, 7 from peripheral blood mononuclear cells (PBMCs), 8 from CD4 ^+^ and/or CD8 ^+^ T-cells, 4 from monocytes, 1 from dendritic cells (DCs), and 7 from tissues different from blood ( Figure 1). Four datasets comprise samples from patients co-infected with tuberculosis (TB) ^7– 10^, one dataset comprises samples from AIDS related lymphomas ^11^, and four datasets addressed HIV infected patients with neurological disorders, such as HIV related fatigue syndrome ^12^, major depression disorder (MDD) ^13^, or HIV-Associated Neurocognitive Disorder (HAND) ^14, 15^. Among the many noteworthy datasets, several stood out, such as the extensive study of the transcriptional signature of early acute HIV infection in whole blood samples of both antiretroviral-treated and untreated populations over the course of infection ^16^ [GXB: GSE29429-GPL10558 and GSE29429-GPL6947]. Several datasets investigate differences in gene expression between distinct stages of HIV infection (early/acute, chronic) ^17, 18^ [GXB: GSE6740, GSE16363], or different host responses to infection (progressors, non-progressors, elite controllers) ^19– 23^ [GXB: GSE28128, GSE24081, GSE56837, GSE23879, GSE18233]. Other studies address different stages or responses to antiretroviral therapy ^24– 26^ [GXB: GSE44228, GSE19087, GSE52900], or transcriptional changes after therapy interruption ^27– 29^ [GXB: GSE10924, GSE28177, GSE5220]. The entirety of the datasets that makes up our collection is listed in Table 1. Thematic composition of our collection is illustrated by a graphical representation of relative occurrences of terms in the list of titles loaded into the GXB tool ( Figure 2).

**Table 1.** List of datasets constituting the collection, also available at http://hiv.gxbsidra.org/dm3/geneBrowser/list.

Title	Platform	Number of samples	Sample source	Validation genes	GEO ID	Ref
Blood Transcriptional Signature of hyperinflammation in HIV-associated Tuberculosis	Illumina HumanHT-12 v4	107	Whole blood	N/A	GSE58411	7
CD4 ^+^ T Cell Decline is Predicted by Differential Expression of Genes in HIV seropositive patients	Affymetrix HG-Focus v1	96	PBMC	N/A	GSE10924	27
CD4 ^+^ T cell gene expression in virologically suppressed HIV-infected patients during Maraviroc intensification therapy	Illumina HumanHT-12 v4	77	CD4 ^+^ T cells	CD3, CD4	GSE56804	30
Chronic CD4 ^+^ T cell Activation and Depletion in HIV-1 Infection: Type I Interferon-Mediated Disruption of T Cell Dynamic	Affymetrix HG-U133_Plus_2	20	CD4 ^+^ T cells	CD3, CD4	GSE9927	31
Comparative analysis of genomic features of human HIV-1 infection and primate models of SIV infection	Illumina HumanWG-6 v3	79	CD4 ^+^ CD8 ^+^ T cells	CD4, CD8	GSE28128	19
Comparison of CD4 ^+^ T cell function between HIV-1 resistant and HIV-1 susceptible individuals (Affymetrix)	Affymetrix HG-U133_Plus_2	18	CD4 ^+^ T cells	CD3, CD4	GSE14278	32
Comparison of gene expression profiles of HIV-specific CD8 T cells from controllers and progressors	Affymetrix HG-U133A	42	CD8 ^+^ T cells	CD8, CD4-neg	GSE24081	20
Comparison of transcriptional profiles of CD4 ^+^ and CD8 ^+^ T cells from HIV-infected patients and uninfected control group	Affymetrix HG-U133A	40	CD4 ^+^ CD8 ^+^ T cells	CD4, CD8	GSE6740	17
Differential Gene Expression in HIV-Infected Individuals Following ART	Illumina HumanWG-6 v3	72	PBMC	XIST	GSE44228	24
Differential Gene Expression of Soluble CD8 ^+^ T-cell mediated suppression of HIV replication in three older children	Affymetrix HG-U133_Plus_2	3	PBMC	XIST	GSE23183	33
Expression data from CD11c+ mDCs in HIV infection	Affymetrix HG-U133_Plus_2	8	mDC	CD11c	GSE42058	34
Expression data from HAART interruption in HIV patients	Affymetrix HG-U133_Plus_2	6	GALT	N/A	GSE28177	28
Expression data from HIV exposed and uninfected women	Affymetrix HG-U133_Plus_2	86	Whole blood	N/A	GSE33580	35
Fatigue-related HIV disease gene-networks identified in CD14 ^+^ cells isolated from HIV-infected patients	Affymetrix FATMITO1a 520158F v1	15	Mono cytes	CD14	GSE18468	12
Gene expression analysis of PBMC from HIV and HIV/TB co-infected patients	Illumina HumanHT-12 v4	44	PBMC	XIST	GSE50834	8
Gene expression before HAART initiation predicts HIV- infected individuals at risk of poor CD4 ^+^ T cell recovery	Illumina HumanWG-6 v3	24	PBMC	XIST	GSE19087	25
Gene Expression in Frontal Cortex in Major Depression and HIV	Affymetrix HG-U133_Plus_2	8	Brain	XIST	GSE17440	13
Gene-expression profiling of HIV-1 infection and perinatal transmission in Botswana	Affymetrix HG-U133A	45	PBMC	N/A	GSE4124	36
Genome wide mRNA expression correlates of viral control in CD4 ^+^T cells from HIV-1 infected individuals	Illumina HumanWG-6 v3	202	CD4 ^+^ T cells	CD3, CD4	GSE18233	23
Genome wide transcriptional profiling of HIV positive and negative children with active tuberculosis, latent TB infection and other diseases	Illumina HumanHT-12 v4	491	Whole blood	N/A	GSE39941 ( GSE39939 + GSE39940)	9
Genome-wide analysis of gene expression in whole blood from HIV-1 progressors and non-progressors	Illumina HumanWG-6 v3	26	Whole blood	N/A	GSE56837	21
Genome-wide transcriptional profiling of HIV positive and negative adults with active tuberculosis, latent TB infection and other diseases - GSE37250_family	Illumina HumanHT-12 v4	537	Whole blood	N/A	GSE37250	10
HIV-1 infection in human PBMCs *in vivo*	Illumina HumanWG-6 v2	87	PBMC	N/A	GSE2171	37
Inflammation and macrophage activation in adipose tissue of HIV-infected patients under antiretroviral treatment	Affymetrix HG-U133A	13	Adipose tissue	ADIPOQ	GSE19811	N/A
Longitudinal comparison of monocytes from an HIV viremic vs avirmeic state	Affymetrix HG-U133A	16	Mono cytes	CD14	GSE5220	29
Microarray Analysis of Lymphatic Tissue Reveals Stage- Specific, Gene-Expression Signatures in HIV-1 Infection	Affymetrix HG-U133_Plus_2	52	Lymph node	XIST	GSE16363	18
Molecular Classification of AIDS-Related Lymphomas	Affymetrix HG-U133_Plus_2	17	Tissues	XIST	GSE17189	11
The National NeuroAIDS Tissue Consortium Brain Gene Array: Two types of HIV-associated neurocognitive impairment	Affymetrix HG-U133_Plus_2	72	Brain	XIST	GSE35864	14
The Relationship between Virus Replication and Host Gene Expression in Lymphatic Tissue during HIV-1 Infection	Affymetrix HG-U133_Plus_2	42	Lymph node	XIST	GSE21589	38
Transcriptional profiling of CD4 T-cells in HIV-1 infected patients	Illumina HumanRef-8 v3	40	CD4 ^+^ T cells	CD3, CD4	GSE23879	22
Transcriptome analysis of HIV-infected peripheral blood monocytes	Illumina HumanHT-12 v4	86	Mono cytes	CD14	GSE50011	15
Transcriptome analysis of primary monocytes from HIV+ patients with differential responses to therapy	Illumina HumanHT-12 v3	14	Mono cytes	CD14	GSE52900	26
Whole Blood Transcriptional Response to Early Acute HIV -GPL10558	Illumina HumanHT-12 v4	47	Whole blood	XIST	GSE29429	16
Whole Blood Transcriptional Response to Early Acute HIV -GPL6947	Illumina HumanHT-12 v3	185	Whole blood	XIST	GSE29429	

Raw data for Figure 1 Click here for additional data file. Copyright: © 2016 Blazkova J et al. 2016 Data associated with the article are available under the terms of the Creative Commons Zero "No rights reserved" data waiver (CC0 1.0 Public domain dedication).

### Gene expression browser (GXB) – dataset upload and annotation

Once a final selection had been made, each dataset was downloaded from GEO as a Simple Omnibus Format in Text (SOFT) file. It was in turn uploaded on a dedicated instance of the GXB, an interactive web application developed at the Benaroya Research Institute, hosted on the Amazon Web Services cloud. Available sample and study information were also uploaded. Samples were grouped according to possible interpretations of study results and gene rankings were computed based on different group comparisons (e.g. comparing samples form HIV negative vs HIV positive patients, with or without antiretroviral therapy, in different stages of disease progression, or with or without co-infection, depending on the focus of respective studies).

### GXB – short tutorial

The GXB software has been described in detail in a recent publication ^5^. This custom software interface provides users with a means to easily navigate and filter the dataset collection available at http://hiv.gxbsidra.org/dm3/geneBrowser/list. A web tutorial is also available online: https://gxb.benaroyaresearch.org/dm3/tutorials.gsp#gxbtut. Briefly, datasets of interest can be quickly identified either by filtering on criteria from pre-defined lists on the left side of the dataset navigation page, or by entering a query term in the search box at the top of the dataset navigation page. Clicking on one of the studies listed in the dataset navigation page opens a viewer designed to provide interactive browsing and graphic representations of large-scale data in an interpretable format. This interface is designed to present ranked gene lists and to display expression results graphically in a context-rich environment. Selecting a gene from the rank-ordered list on the left of the data-viewing interface will display its expression values graphically in the screen’s central panel. Directly above the graphical display, drop down menus give users the ability: a) To change the rank list by selecting different comparisons (in cases where the dataset is split in more than two groups), or to only include genes that are selected for specific biological interest. b) To change sample grouping (Group Set button); in some datasets, user can switch between interpretations where samples are grouped based on cell type or disease, for example. c) To sort individual samples within a group based on associated categorical or continuous variables (e.g. gender or age). d) To toggle between a bar plot view and a box plot view, with expression values represented as a single point for each sample. Samples are split into the same groups whether displayed as a bar plot or a box plot. e) To provide a color legend for the sample groups. f) To select categorical information to be overlaid at the bottom of the graph. For example, the user can display gender or smoking status in this manner. g) To provide a color legend for the categorical information overlaid at the bottom of the graph. h) To download the graph as a portable network graphics (png) image or the table with expression values as a comma separated values (csv) file. Measurements have no intrinsic utility in absence of contextual information. It is this contextual information that makes the results of a study or experiment interpretable. It is therefore important to capture, integrate and display information that will give users the ability to interpret data and gain new insights from it. We have organized this information under different tabs directly above the graphical display. The tabs can be hidden to make more room for displaying the data plots, or revealed by clicking on the blue “hide/show info panel” button on the top right corner of the display. Information about the gene selected from the list on the left side of the display is available under the “Gene” tab. Information about the study is available under the “Study” tab. Information available about individual samples is provided under the “Sample” tab. Rolling the mouse cursor over a bar plot, while displaying the “Sample” tab, lists any clinical, demographic, or laboratory information available for the selected sample. Finally, the “Downloads” tab allows advanced users to retrieve the original dataset for analysis outside this tool. It also provides all available sample annotation data for use alongside the expression data in third party analysis software. Other functionalities are provided under the “Tools” drop-down menu located in the top right corner of the user interface. These functionalities include notably: a) “Annotations”, which provides access to all the ancillary information about the study, samples and the dataset, organized across different tabs; b) “Cross Project View”, which provides the ability to browse across all available studies for a given gene; c) “Copy Link”, which generates a mini-URL encapsulating information about the display settings in use and that can be saved and shared with others (clicking on the envelope icon on the toolbar inserts the url in an email message via the local email client); and d) “Chart Options”, which gives user the option to customize chart labels.

### Dataset validation

Quality control checks were performed by examination of profiles of relevant biological markers. Known leukocyte surface markers were used to verify consistency of the information provided by dataset depositors, and to identify instances where contamination of samples by other leukocyte populations may be confounding. The markers that were used include: CD3 (CD3D), a T-cell marker; CD4 and CD8 (CD8A), markers of CD4 ^+^ and CD8 ^+^ T cells respectively; CD11c (ITGAX), an mDC marker; CD14, expressed by monocytes and macrophages; or Adiponectin (ADIPOQ), expressed in adipose tissue. Expression of the XIST transcripts, which expression is gender-specific, was also examined in datasets containing relevant information, to determine its concordance with demographic information provided with the GEO submission (respective links in Table 1).

## Data availability

The data referenced by this article are under copyright with the following copyright statement: Copyright: © 2016 Blazkova J et al.

Data associated with the article are available under the terms of the Creative Commons Zero "No rights reserved" data waiver (CC0 1.0 Public domain dedication). 

All datasets included in our curated collection are also available publically via the NCBI GEO website: www.ncbi.gov/geo; and are referenced throughout the manuscript by their GEO accession numbers (e.g. GSE44228). Signal files and sample description files can also be downloaded from the GXB tool under the “downloads” tab.

*F1000Research*: Dataset 1. Raw data for Figure 1, 10.5256/f1000research.8204.d115581 ^39^
