# Supplementary material for: A curated transcriptome dataset collection to investigate the immunobiology of HIV infection
Source: F1000Res. 2016 Mar 11;5:327. [Version 1] doi: 10.12688/f1000research.8204.1 (PMC4838008; doi:10.12688/f1000research.8204.1)
Supplement: Peer review history

# Peer review: Referee response for version 1

**DOI:** 10.5256/f1000research.8824.r12871

**Published:** 2016-04-20

**Authors:** José Alcami, Francisco Diez-Fuertes

**Affiliations:** 1 Centro Nacional de Microbiologia, Instituto de Salud Carlos III, Majadahonda, Madrid, Spain; 2 Instituto de Salud Carlos III, Majadahonda, Madrid, Spain

Blazkova *et al. *describe an interactive web application that includes 34 different transcriptome datasets. This open tool facilitates access to transcriptome analysis in the HIV field allowing meta-analyses on transcriptomic changes in HIV infection.

As strengths of the article I will highlight:

- The application is friendly and easy to use and allowed us to compare our results with a large collection of databases in a comprehensive way.
- The software allows searches related with a particular gene and how its expression is modified in different scenarios (infected vs non-infected, long term non-progressors vs typical progressors, treated vs untreated). 
- The cellular types in which dataset have been obtained are indicated.
- Datasets included have been selected according to their interest and high methodological standards. For example, when contamination with cell types different from those initially  targeted are detected the studies are not considered for the final dataset thus enhancing the quality of the results. 

I would propose some suggestions to improve this interesting tool:

- All the studies were performed with microarrays. It would be important to discuss if the inclusion of data using RNA-seq approaches and the current units used in these studies (FPKMs, RPKMs,TPMs) could be incorporated in the future.
- It should be clarified if the results among the different studies are normalized or just described with the units used in each study. If data normalization has been performed it would important to describe how it was done. 

Overall it represents an important effort that can be useful for many researchers working in the field of HIV genetics and pathogenesis.

We have read this submission. We believe that we have an appropriate level of expertise to confirm that it is of an acceptable scientific standard.

# Peer review: Referee response for version 1

**DOI:** 10.5256/f1000research.8824.r13356

**Published:** 2016-04-15

**Authors:** Nicolas Chomont

**Affiliations:** 1 Department of Microbiology, Infectiology and Immunology, Université de Montréal, Montreal, QC, Canada

In this interesting article, Blazkova and colleagues describe the development of an interactive web application that allows HIV researchers to access a collection of transcriptome datasets relevant to HIV infection. The collection includes 34 datasets generated with human samples that have been carefully selected based on their relevance and quality control checks.

This is a very useful tool that can be easily used by non-experts in transcriptomics analyses. I have used it and I am convinced that it potentially represents an important contribution to the work performed by HIV researchers. I tested the accuracy of the tool (not in a formal way) by examining differences in the expression for several genes that are well-known to be modulated by HIV infection. The results are clearly presented and can be easily exported to be included in presentations/publications.

A few suggestions: I anticipate that the database will be updated on a regular basis. Therefore, it would be great to specify the date of the last update of the data set available online. Also, the possibility of adding RNAseq data would be important in the future. Maybe a brief description of each dataset would be useful too.

I have read this submission. I believe that I have an appropriate level of expertise to confirm that it is of an acceptable scientific standard.

# Peer review: Referee response for version 1

**DOI:** 10.5256/f1000research.8824.r12874

**Published:** 2016-04-12

**Authors:** Amalio Telenti

**Affiliations:** 1 J. Craig Venter Institute (JCVI), La Jolla, CA, USA

The article by Blazkova and colleagues constitutes an important contribution to the HIV field. It crystallizes the efforts of multiple groups that characterized the host transcriptional response to infection by providing a viewer of data that are not immediately accessible in a structured interface. I have assessed the performance of the tool, and found it intuitive and user-friendly.

It extends efforts of my group to provide facilitated access to gnomic data in HIV disease ( http://www.guavah.org/).

I would bring two aspects up for discussion. First, that this tool should evolve to display RNAseq data = new generation sequencing data are increasingly available, effectively displacing microarrays. RNAseq is also easier for standardization across studies. Second, that users should be attentive to the subtleties of analysis: covariates such as gender, age, cellularity, analytical platforms and batch effects can influence expression profiles significantly. In-depth analysis may thus require downloading of original expression data.

I have read this submission. I believe that I have an appropriate level of expertise to confirm that it is of an acceptable scientific standard.
